# 9-(4-Methyl­phenoxy­carbon­yl)-10-methyl­acridinium trifluoro­methane­sulfonate

**DOI:** 10.1107/S1600536810016302

**Published:** 2010-05-12

**Authors:** Damian Trzybiński, Karol Krzymiński, Artur Sikorski, Jerzy Błażejowski

**Affiliations:** a Faculty of Chemistry, University of Gdańsk, J. Sobieskiego 18, 80-952 Gdańsk, Poland

## Abstract

In the crystal structure of the title compound, C_22_H_18_NO_2_^+^·CF_3_SO_3_^−^, adjacent cations are linked through C—H⋯π and π–π inter­actions, and the cations and anions are connected by C—H⋯O and C—F⋯π inter­actions. The acridine and benzene ring systems are oriented at a dihedral angle of 3.0 (1)°. The carboxyl group is twisted at an angle of 83.1 (1)° relative to the acridine skeleton. The mean planes of adjacent acridine units are parallel or inclined at an angle of 75.2 (1)° in the crystal structure.

## Related literature

For background to the chemiluminogenic properties of 9-phenoxy­carbonyl-10-methyl­acridinium trifluoro­methane­sulf­onates, see: Brown *et al.* (2009 ▶); Rak *et al.* (1999 ▶); Roda *et al.* (2003 ▶); Zomer & Jacquemijns (2001 ▶). For related structures, see: Sikorski *et al.* (2006 ▶); Trzybiński *et al.* (2010 ▶). For inter­molecular inter­actions, see: Bianchi *et al.* (2004 ▶); Dorn *et al.* (2005 ▶); Hunter *et al.* (2001 ▶); Novoa *et al.* (2006 ▶); Takahashi *et al.* (2001 ▶). For the synthesis, see: Sato (1996 ▶); Sikorski *et al.* (2006 ▶); Trzybiński *et al.* (2010 ▶).
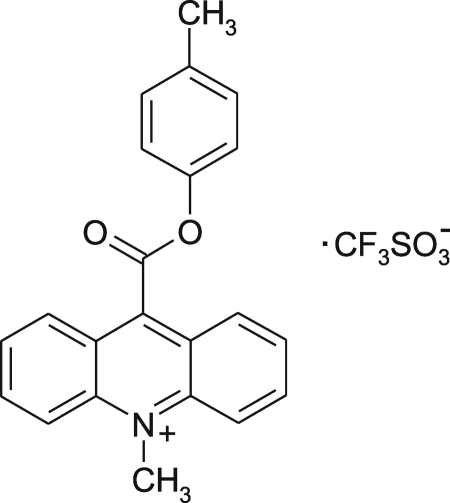


## Experimental

### 

#### Crystal data

C_22_H_18_NO_2_^+^·CF_3_O_3_S^−^
*M* *_r_* = 477.45
Monoclinic, 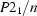

*a* = 13.2686 (6) Å
*b* = 8.4788 (4) Å
*c* = 20.4078 (10) Å
β = 106.749 (5)°
*V* = 2198.51 (19) Å^3^
*Z* = 4
Mo *K*α radiation
μ = 0.21 mm^−1^
*T* = 295 K
0.50 × 0.40 × 0.10 mm

#### Data collection

Oxford Diffraction Gemini R Ultra Ruby CCD diffractometer
Absorption correction: multi-scan (*CrysAlis RED*; Oxford Diffraction, 2008 ▶) *T*_min_ = 0.869, *T*_max_ = 1.000
12172 measured reflections
3892 independent reflections
2096 reflections with *I* > 2σ(*I*)
*R*_int_ = 0.061

#### Refinement

*R*[*F* ^2^ > 2σ(*F* ^2^)] = 0.057
*wR*(*F* ^2^) = 0.137
*S* = 0.95
3892 reflections
299 parameters
H-atom parameters constrained
Δρ_max_ = 0.23 e Å^−3^
Δρ_min_ = −0.19 e Å^−3^

### 

Data collection: *CrysAlis CCD* (Oxford Diffraction, 2008 ▶); cell refinement: *CrysAlis RED*; data reduction: *CrysAlis RED*; program(s) used to solve structure: *SHELXS97* (Sheldrick, 2008 ▶); program(s) used to refine structure: *SHELXL97* (Sheldrick, 2008 ▶); molecular graphics: *ORTEP-3* (Farrugia, 1997 ▶); software used to prepare material for publication: *SHELXL97* and *PLATON* (Spek, 2009 ▶).

## Supplementary Material

Crystal structure: contains datablocks global, I. DOI: 10.1107/S1600536810016302/ng2760sup1.cif

Structure factors: contains datablocks I. DOI: 10.1107/S1600536810016302/ng2760Isup2.hkl

Additional supplementary materials:  crystallographic information; 3D view; checkCIF report

## Figures and Tables

**Table 1:** Hydrogen-bond geometry (Å, °) *Cg*4 is the centroid of the C18–C23 ring.

*D*—H⋯*A*	*D*—H	H⋯*A*	*D*⋯*A*	*D*—H⋯*A*
C3—H3⋯O27^i^	0.93	2.57	3.314 (5)	137
C4—H4⋯O29^i^	0.93	2.44	3.319 (4)	159
C5—H5⋯O28	0.93	2.44	3.364 (5)	171
C6—H6⋯O28^ii^	0.93	2.56	3.342 (5)	142
C23—H23⋯O27^iii^	0.93	2.53	3.448 (4)	169
C25—H25*A*⋯O29	0.96	2.56	3.415 (5)	149
C25—H25*B*⋯*Cg*4^iv^	0.96	2.62	3.487 (4)	151

Symmetry codes: (i) 
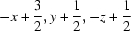
; (ii) 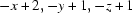; (iii) 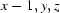; (iv) 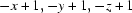.

**Table 2:** C–F⋯π inter­actions (Å,°) *Cg*1 and *Cg*2 are the centroids of the C9/N10/C11–C14 and C1–C4/C11/C12 rings, respectively.

*X*	*I*	*J*	*I*⋯*J*	*X*⋯*J*	*X*–*I*⋯*J*
C30	F31	*Cg*2^i^	3.420 (3)	4.044 (4)	108.9 (2)
C30	F32	*Cg*1^i^	3.441 (3)	4.032 (4)	107.1 (2)
C30	F32	*Cg*2^i^	3.788 (4)	4.044 (4)	91.5 (2)
C30	F33	*Cg*1^i^	3.669 (3)	4.032 (4)	96.2 (2)

Symmetry code: (i) 
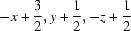
.

**Table 3:** π–π inter­actions (Å,°) *Cg*3 and *Cg*4 are the centroids of the C5–C8/C13/C14 and C18–C23 rings, respectively. *CgI*⋯*CgJ* is the distance between ring centroids. The dihedral angle is that between the planes of the rings *I* and *J. CgI*_Perp is the perpendicular distance of *CgI* from ring *J. CgI*_Offset is the distance between *CgI* and perpendicular projection of *CgJ* on ring *I*.

*I*	*J*	*CgI*⋯*CgJ*	Dihedral angle	*CgI*_Perp	*CgI*_Offset
3	4^v^	3.913 (2)	4.80 (17)	3.472 (2)	1.805 (2)
4	3^v^	3.913 (2)	4.80 (17)	3.565 (2)	1.613 (2)

Symmetry code: (v) 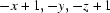.

**Fig. 1.**
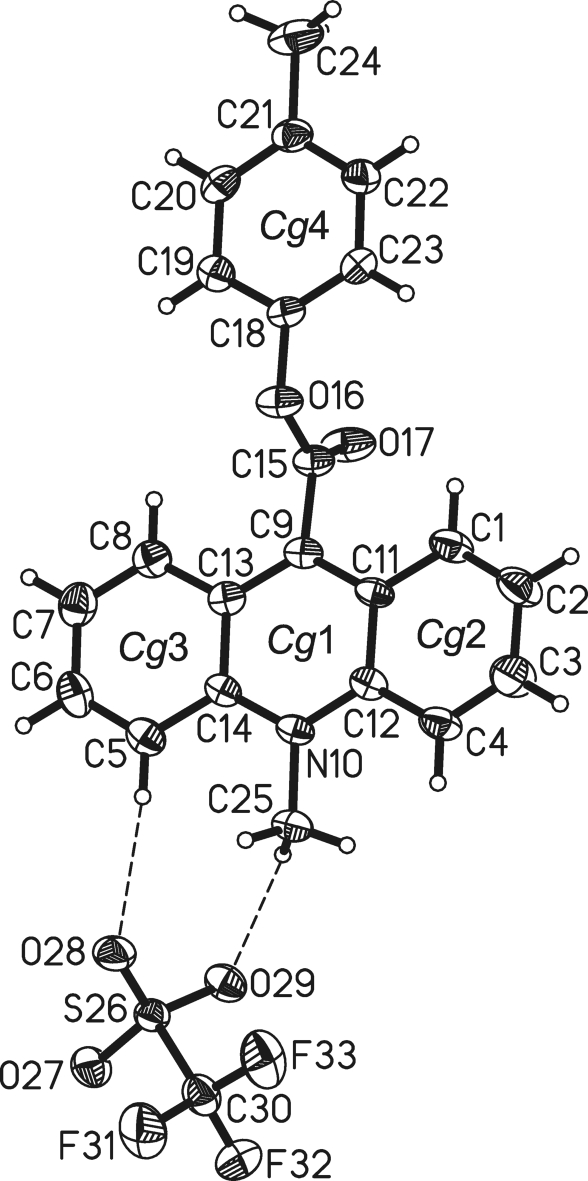
The molecular structure of the title compound showing the atom-labeling scheme. Displacement ellipsoids are drawn at the 25% probability level and H atoms are shown as small spheres of arbitrary radius. Cg1, Cg2, Cg3 and Cg4 denote the ring centroids. The C–H···O hydrogen bonds are represented by dashed lines.

**Fig. 2.**
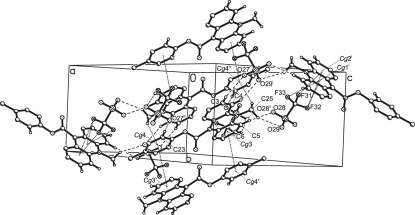
The arrangement of the ions in the crystal structure. The C–H···O interactions are represented by dashed lines, the C–H···π, C–F···π and π–π contacts by dotted lines. H atoms not involved in interactions have been omitted. [Symmetry codes: (i) –x + 3/2, y + 1/2, –z + 1/2; (ii) –x + 2, –y + 1, –z + 1; (iii) x – 1, y, z; (iv) –x + 1, –y + 1, –z + 1; (v) –x + 1, –y, –z + 1.]

## supplementary crystallographic information

### Comment

9-(Phenoxycarbonyl)-10-methylacridinium salts appear to be convenient chemiluminescent indicators or the chemiluminogenic fragments of chemiluminescent labels (Zomer & Jacquemijns, 2001), which are widely applied in assays of biologically and environmentally important entities such as antigens, antibodies, enzymes or DNA fragments (Roda *et al.*, 2003; Brown *et al.*, 2009). Oxidation of the cations of these salts with hydrogen peroxide in alkaline media is accompanied by the removal of the phenoxycarbonyl fragment and the conversion of the remaining part of the molecule to electronically excited, light emitting 10-methyl-9-acridinone (Rak *et al.*, 1999; Zomer & Jacquemijns, 2001). The efficiency of chemiluminescence – crucial for analytical applications – is affected by the constitution of the phenyl fragment (Zomer & Jacquemijns, 2001). This prompted us to undertake investigations on derivatives substituted in this fragment. Here we present the structure of 9-(4-methylphenoxy)carbonyl-10-methylacridinium trifluoromethanesulfonate, a structural isomer of the 2-methyl substituted salt, whose structure has already been refined (Sikorski *et al.*, 2006).

In the cation of the title compound (Fig. 1), the bond lengths and angles characterizing the geometry of the acridinium moiety are typical of acridine-based derivatives (Sikorski *et al.*, 2006; Trzybiński *et al.*, 2010). With respective average deviations from planarity of 0.0386 (3) Å and 0.0017 (3) Å, the acridine and benzene ring systems are oriented at a dihedral angle of 3.0 (1)°. The carboxyl group is twisted at an angle of 83.1 (1)° relative to the acridine skeleton. The mean planes of the adjacent acridine moieties are parallel (remain at an angle of 0.0 (1)°) or inclined at an angle of 75.2 (1)° in the lattice.

In the crystal structure, the adjacent cations are linked through C–H···π (Table 1, Fig. 2) and π–π (involving acridine and phenyl moieties) (Table 3, Fig. 2) interactions, and cations and anions are connected by multidirectional C–H···O (Table 1, Figs. 1 and 2) and C–F···π (Table 2, Fig. 2) interactions. The C–H···O interactions are of the hydrogen bond type (Bianchi *et al.* 2004; Novoa *et al.*, 2006). The C–H···π interactions should be of an attractive nature (Takahashi *et al.*, 2001), like the C–F···π (Dorn *et al.*, 2005) and π–π (Hunter *et al.*, 2001) interactions. The crystal structure is stabilized by a network of these short-range specific interactions and by long-range electrostatic interactions between ions.

### Experimental

The compound was synthesized as described elsewhere (Sikorski *et al.*, 2006; Trzybiński *et al.*, 2010), i.e., 9-(chlorocarbonyl)acridine obtained by treating acridine-9-carboxylic acid with a tenfold molar excess of thionyl chloride was esterified with 4-methylphenol in anhydrous dichloromethane in the presence of *N*,*N*-diethylethanamine and a catalytic amount of *N*,*N*-dimethyl-4-pyridinamine (room temperature, 15h) (Sato, 1996). The product – 4-methylphenyl acridine-9-carboxylate, purified chromatographically (SiO_2_, cyclohexane/ethyl acetate, 3/2 v/v) – was quaternarized with a fivefold molar excess of methyl trifluoromethanesulfonate dissolved in anhydrous dichloromethane. The crude 9-(4-methylphenoxycarbonyl)-10-methylacridinium trifluoromethanesulfonate was dissolved in a small amount of ethanol, filtered and precipitated with 25 v/v excess of diethyl ether. Light-orange crystals suitable for X-ray investigations were grown from absolute ethanol solution (m.p. 445 - 447 K).

### Refinement

H atoms were positioned geometrically, with C—H = 0.93 Å and 0.96 Å for the aromatic and methyl H atoms, respectively, and constrained to ride on their parent atoms with *U*_iso_(H) = *xU*_eq_(C), where *x* = 1.2 for the aromatic and *x* = 1.5 for the methyl H atoms.

### Crystal data

**Table:**

C_22_H_18_NO_2_^+^·CF_3_O_3_S^−^	*F*(000) = 984
*M**_r_* = 477.45	*D*_x_ = 1.442 Mg m^−^^3^
Monoclinic, *P*2_1_/*n*	Mo *K*α radiation, λ = 0.71073 Å
Hall symbol: -P 2yn	Cell parameters from 3355 reflections
*a* = 13.2686 (6) Å	θ = 3.1–29.2°
*b* = 8.4788 (4) Å	µ = 0.21 mm^−^^1^
*c* = 20.4078 (10) Å	*T* = 295 K
β = 106.749 (5)°	Plate, light-orange
*V* = 2198.51 (19) Å^3^	0.50 × 0.40 × 0.10 mm
*Z* = 4	

### Data collection

**Table:**

Oxford Diffraction Gemini R Ultra Ruby CCD diffractometer	3892 independent reflections
Radiation source: enhanced (Mo) X-Ray Source	2096 reflections with *I* > 2σ(*I*)
graphite	*R*_int_ = 0.061
Detector resolution: 10.4002 pixels mm^-1^	θ_max_ = 25.1°, θ_min_ = 3.1°
ω scans	*h* = −15→15
Absorption correction: multi-scan (*CrysAlis RED*; Oxford Diffraction, 2008)	*k* = −10→10
*T*_min_ = 0.869, *T*_max_ = 1.000	*l* = −24→24
12172 measured reflections	

### Refinement

**Table:**

Refinement on *F*^2^	Primary atom site location: structure-invariant direct methods
Least-squares matrix: full	Secondary atom site location: difference Fourier map
*R*[*F*^2^ > 2σ(*F*^2^)] = 0.057	Hydrogen site location: inferred from neighbouring sites
*wR*(*F*^2^) = 0.137	H-atom parameters constrained
*S* = 0.95	*w* = 1/[σ^2^(*F*_o_^2^) + (0.0678*P*)^2^] where *P* = (*F*_o_^2^ + 2*F*_c_^2^)/3
3892 reflections	(Δ/σ)_max_ < 0.001
299 parameters	Δρ_max_ = 0.23 e Å^−^^3^
0 restraints	Δρ_min_ = −0.19 e Å^−^^3^

### Special details

**Table:**

Geometry. All esds (except the esd in the dihedral angle between two l.s. planes) are estimated using the full covariance matrix. The cell esds are taken into account individually in the estimation of esds in distances, angles and torsion angles; correlations between esds in cell parameters are only used when they are defined by crystal symmetry. An approximate (isotropic) treatment of cell esds is used for estimating esds involving l.s. planes.

### Fractional atomic coordinates and isotropic or equivalent isotropic displacement parameters (Å^2^)

**Table:**

	*x*	*y*	*z*	*U*_iso_*/*U*_eq_	
C1	0.3367 (3)	0.4502 (4)	0.3326 (2)	0.0699 (10)	
H1	0.2898	0.4002	0.3519	0.084*	
C2	0.2997 (3)	0.5409 (5)	0.2766 (2)	0.0918 (13)	
H2	0.2276	0.5502	0.2563	0.110*	
C3	0.3705 (3)	0.6211 (5)	0.2491 (2)	0.0957 (14)	
H3	0.3441	0.6854	0.2111	0.115*	
C4	0.4760 (3)	0.6083 (4)	0.2759 (2)	0.0691 (10)	
H4	0.5208	0.6634	0.2565	0.083*	
C5	0.7739 (3)	0.3924 (4)	0.44967 (19)	0.0631 (9)	
H5	0.8203	0.4433	0.4302	0.076*	
C6	0.8115 (3)	0.3064 (5)	0.5083 (2)	0.0735 (10)	
H6	0.8838	0.3013	0.5287	0.088*	
C7	0.7440 (3)	0.2253 (5)	0.5386 (2)	0.0766 (11)	
H7	0.7713	0.1667	0.5783	0.092*	
C8	0.6389 (3)	0.2334 (4)	0.50934 (18)	0.0639 (10)	
H8	0.5944	0.1786	0.5291	0.077*	
C9	0.4876 (3)	0.3337 (4)	0.41922 (16)	0.0479 (8)	
N10	0.62352 (19)	0.4910 (3)	0.36094 (13)	0.0486 (7)	
C11	0.4461 (2)	0.4303 (4)	0.36217 (16)	0.0498 (8)	
C12	0.5177 (2)	0.5109 (4)	0.33342 (16)	0.0495 (8)	
C13	0.5948 (3)	0.3229 (4)	0.44951 (16)	0.0507 (8)	
C14	0.6651 (2)	0.4037 (4)	0.41908 (16)	0.0473 (8)	
C15	0.4137 (3)	0.2312 (4)	0.44360 (17)	0.0548 (9)	
O16	0.38896 (18)	0.2932 (3)	0.49667 (12)	0.0648 (7)	
O17	0.3828 (2)	0.1080 (3)	0.41764 (13)	0.0858 (9)	
C18	0.3185 (3)	0.2065 (4)	0.52390 (16)	0.0500 (8)	
C19	0.3597 (3)	0.1129 (4)	0.57992 (17)	0.0609 (9)	
H19	0.4321	0.0997	0.5978	0.073*	
C20	0.2903 (3)	0.0381 (4)	0.60930 (17)	0.0652 (10)	
H20	0.3170	−0.0262	0.6473	0.078*	
C21	0.1839 (3)	0.0565 (4)	0.58395 (19)	0.0647 (10)	
C22	0.1461 (3)	0.1515 (5)	0.52795 (19)	0.0671 (10)	
H22	0.0739	0.1655	0.5102	0.081*	
C23	0.2132 (3)	0.2273 (4)	0.49707 (17)	0.0593 (9)	
H23	0.1866	0.2908	0.4589	0.071*	
C24	0.1088 (4)	−0.0241 (5)	0.6176 (2)	0.1041 (15)	
H24A	0.0428	0.0309	0.6055	0.156*	
H24B	0.1387	−0.0225	0.6664	0.156*	
H24C	0.0978	−0.1313	0.6021	0.156*	
C25	0.6972 (3)	0.5623 (4)	0.32692 (17)	0.0655 (10)	
H25A	0.7583	0.4964	0.3343	0.098*	
H25B	0.7180	0.6650	0.3458	0.098*	
H25C	0.6632	0.5715	0.2787	0.098*	
S26	0.98783 (7)	0.47984 (11)	0.33273 (4)	0.0560 (3)	
O27	1.09157 (18)	0.4193 (3)	0.34606 (12)	0.0801 (8)	
O28	0.96339 (18)	0.5485 (3)	0.39004 (12)	0.0844 (8)	
O29	0.90554 (18)	0.3856 (3)	0.28984 (12)	0.0688 (7)	
C30	0.9911 (3)	0.6484 (5)	0.2789 (2)	0.0737 (11)	
F31	1.0614 (2)	0.7536 (3)	0.31098 (15)	0.1247 (10)	
F32	1.0156 (2)	0.6073 (4)	0.22295 (13)	0.1123 (9)	
F33	0.8988 (2)	0.7204 (3)	0.25876 (13)	0.1117 (9)	

### Atomic displacement parameters (Å^2^)

**Table:**

	*U*^11^	*U*^22^	*U*^33^	*U*^12^	*U*^13^	*U*^23^
C1	0.057 (2)	0.061 (2)	0.098 (3)	−0.003 (2)	0.033 (2)	0.014 (2)
C2	0.050 (2)	0.096 (3)	0.131 (4)	0.012 (2)	0.029 (2)	0.033 (3)
C3	0.078 (3)	0.090 (3)	0.117 (3)	0.012 (3)	0.027 (3)	0.046 (3)
C4	0.056 (2)	0.060 (2)	0.098 (3)	−0.0012 (19)	0.032 (2)	0.019 (2)
C5	0.060 (2)	0.052 (2)	0.079 (3)	−0.0077 (19)	0.022 (2)	−0.011 (2)
C6	0.061 (2)	0.063 (3)	0.085 (3)	−0.001 (2)	0.003 (2)	−0.010 (2)
C7	0.086 (3)	0.063 (3)	0.073 (3)	−0.001 (2)	0.011 (2)	0.004 (2)
C8	0.071 (3)	0.054 (2)	0.067 (2)	−0.008 (2)	0.020 (2)	−0.003 (2)
C9	0.058 (2)	0.0330 (18)	0.060 (2)	−0.0060 (16)	0.0285 (18)	−0.0113 (17)
N10	0.0474 (17)	0.0375 (15)	0.0676 (17)	−0.0061 (13)	0.0274 (14)	−0.0070 (14)
C11	0.048 (2)	0.0370 (18)	0.071 (2)	−0.0068 (16)	0.0289 (18)	−0.0049 (18)
C12	0.052 (2)	0.0331 (19)	0.067 (2)	−0.0010 (16)	0.0229 (17)	−0.0037 (17)
C13	0.063 (2)	0.0320 (18)	0.060 (2)	−0.0040 (17)	0.0225 (18)	−0.0059 (16)
C14	0.046 (2)	0.0351 (18)	0.063 (2)	−0.0052 (15)	0.0203 (17)	−0.0105 (17)
C15	0.068 (2)	0.040 (2)	0.066 (2)	−0.0062 (18)	0.0334 (19)	−0.0037 (18)
O16	0.0833 (17)	0.0462 (14)	0.0804 (16)	−0.0143 (13)	0.0485 (14)	−0.0130 (12)
O17	0.122 (2)	0.0581 (17)	0.1013 (19)	−0.0380 (16)	0.0701 (18)	−0.0252 (16)
C18	0.066 (2)	0.0374 (19)	0.054 (2)	−0.0074 (18)	0.0285 (19)	−0.0066 (17)
C19	0.061 (2)	0.057 (2)	0.066 (2)	−0.0032 (19)	0.0198 (19)	−0.008 (2)
C20	0.091 (3)	0.054 (2)	0.0529 (19)	−0.004 (2)	0.024 (2)	0.0038 (18)
C21	0.074 (3)	0.068 (3)	0.060 (2)	−0.014 (2)	0.032 (2)	−0.014 (2)
C22	0.061 (2)	0.078 (3)	0.068 (2)	−0.001 (2)	0.027 (2)	−0.004 (2)
C23	0.072 (3)	0.055 (2)	0.053 (2)	0.002 (2)	0.0214 (19)	0.0023 (17)
C24	0.141 (4)	0.097 (3)	0.099 (3)	−0.038 (3)	0.074 (3)	−0.008 (3)
C25	0.058 (2)	0.068 (2)	0.079 (2)	−0.0098 (19)	0.0331 (19)	−0.0052 (19)
S26	0.0511 (5)	0.0592 (6)	0.0605 (5)	0.0019 (5)	0.0208 (4)	0.0024 (5)
O27	0.0581 (15)	0.0891 (19)	0.0933 (18)	0.0225 (14)	0.0221 (13)	0.0194 (15)
O28	0.0716 (16)	0.118 (2)	0.0732 (16)	−0.0188 (16)	0.0366 (14)	−0.0244 (15)
O29	0.0627 (15)	0.0584 (15)	0.0878 (17)	−0.0099 (12)	0.0258 (13)	−0.0128 (13)
C30	0.062 (3)	0.066 (3)	0.087 (3)	−0.012 (2)	0.011 (2)	0.000 (2)
F31	0.122 (2)	0.0878 (19)	0.140 (2)	−0.0465 (17)	0.0005 (17)	0.0175 (16)
F32	0.114 (2)	0.147 (2)	0.0809 (15)	−0.0172 (17)	0.0356 (14)	0.0272 (16)
F33	0.0992 (18)	0.0718 (16)	0.142 (2)	0.0200 (15)	−0.0010 (16)	0.0185 (15)

### Geometric parameters (Å, °)

**Table:**

C1—C2	1.347 (5)	C15—O16	1.327 (4)
C1—C11	1.412 (4)	O16—C18	1.421 (3)
C1—H1	0.9300	C18—C23	1.357 (4)
C2—C3	1.401 (5)	C18—C19	1.370 (4)
C2—H2	0.9300	C19—C20	1.388 (5)
C3—C4	1.352 (5)	C19—H19	0.9300
C3—H3	0.9300	C20—C21	1.366 (5)
C4—C12	1.411 (5)	C20—H20	0.9300
C4—H4	0.9300	C21—C22	1.369 (5)
C5—C6	1.366 (5)	C21—C24	1.524 (5)
C5—C14	1.401 (4)	C22—C23	1.388 (4)
C5—H5	0.9300	C22—H22	0.9300
C6—C7	1.406 (5)	C23—H23	0.9300
C6—H6	0.9300	C24—H24A	0.9600
C7—C8	1.351 (5)	C24—H24B	0.9600
C7—H7	0.9300	C24—H24C	0.9600
C8—C13	1.413 (4)	C25—H25A	0.9600
C8—H8	0.9300	C25—H25B	0.9600
C9—C13	1.381 (4)	C25—H25C	0.9600
C9—C11	1.400 (4)	S26—O27	1.420 (2)
C9—C15	1.499 (4)	S26—O28	1.425 (2)
N10—C12	1.364 (4)	S26—O29	1.430 (2)
N10—C14	1.372 (4)	S26—C30	1.811 (4)
N10—C25	1.481 (4)	C30—F32	1.321 (4)
C11—C12	1.427 (4)	C30—F31	1.321 (4)
C13—C14	1.434 (4)	C30—F33	1.324 (4)
C15—O17	1.189 (4)		
			
C2—C1—C11	120.7 (3)	O16—C15—C9	112.4 (3)
C2—C1—H1	119.7	C15—O16—C18	117.3 (2)
C11—C1—H1	119.7	C23—C18—C19	121.9 (3)
C1—C2—C3	119.5 (4)	C23—C18—O16	119.4 (3)
C1—C2—H2	120.2	C19—C18—O16	118.5 (3)
C3—C2—H2	120.2	C18—C19—C20	118.1 (3)
C4—C3—C2	122.3 (4)	C18—C19—H19	121.0
C4—C3—H3	118.9	C20—C19—H19	121.0
C2—C3—H3	118.9	C21—C20—C19	121.8 (3)
C3—C4—C12	119.7 (3)	C21—C20—H20	119.1
C3—C4—H4	120.1	C19—C20—H20	119.1
C12—C4—H4	120.1	C20—C21—C22	118.2 (3)
C6—C5—C14	119.7 (3)	C20—C21—C24	121.1 (4)
C6—C5—H5	120.1	C22—C21—C24	120.7 (4)
C14—C5—H5	120.1	C21—C22—C23	121.5 (3)
C5—C6—C7	121.8 (4)	C21—C22—H22	119.2
C5—C6—H6	119.1	C23—C22—H22	119.2
C7—C6—H6	119.1	C18—C23—C22	118.5 (3)
C8—C7—C6	119.2 (4)	C18—C23—H23	120.7
C8—C7—H7	120.4	C22—C23—H23	120.7
C6—C7—H7	120.4	C21—C24—H24A	109.5
C7—C8—C13	121.7 (3)	C21—C24—H24B	109.5
C7—C8—H8	119.1	H24A—C24—H24B	109.5
C13—C8—H8	119.1	C21—C24—H24C	109.5
C13—C9—C11	121.2 (3)	H24A—C24—H24C	109.5
C13—C9—C15	120.1 (3)	H24B—C24—H24C	109.5
C11—C9—C15	118.5 (3)	N10—C25—H25A	109.4
C12—N10—C14	122.2 (2)	N10—C25—H25B	109.5
C12—N10—C25	119.8 (3)	H25A—C25—H25B	109.5
C14—N10—C25	118.0 (3)	N10—C25—H25C	109.6
C9—C11—C1	122.4 (3)	H25A—C25—H25C	109.5
C9—C11—C12	118.2 (3)	H25B—C25—H25C	109.5
C1—C11—C12	119.4 (3)	O27—S26—O28	115.33 (16)
N10—C12—C4	121.7 (3)	O27—S26—O29	116.23 (16)
N10—C12—C11	120.0 (3)	O28—S26—O29	114.60 (14)
C4—C12—C11	118.3 (3)	O27—S26—C30	102.13 (17)
C9—C13—C8	122.6 (3)	O28—S26—C30	103.11 (19)
C9—C13—C14	119.3 (3)	O29—S26—C30	102.54 (16)
C8—C13—C14	118.2 (3)	F32—C30—F31	107.0 (3)
N10—C14—C5	121.9 (3)	F32—C30—F33	106.9 (3)
N10—C14—C13	118.9 (3)	F31—C30—F33	107.5 (3)
C5—C14—C13	119.3 (3)	F32—C30—S26	111.8 (3)
O17—C15—O16	125.2 (3)	F31—C30—S26	111.7 (3)
O17—C15—C9	122.4 (3)	F33—C30—S26	111.7 (3)
			
C11—C1—C2—C3	−2.5 (6)	C6—C5—C14—C13	−0.7 (5)
C1—C2—C3—C4	1.5 (7)	C9—C13—C14—N10	0.7 (4)
C2—C3—C4—C12	0.2 (6)	C8—C13—C14—N10	180.0 (3)
C14—C5—C6—C7	1.3 (5)	C9—C13—C14—C5	−179.8 (3)
C5—C6—C7—C8	−0.5 (5)	C8—C13—C14—C5	−0.5 (4)
C6—C7—C8—C13	−0.8 (5)	C13—C9—C15—O17	−94.0 (4)
C13—C9—C11—C1	−176.4 (3)	C11—C9—C15—O17	81.1 (4)
C15—C9—C11—C1	8.5 (4)	C13—C9—C15—O16	85.3 (3)
C13—C9—C11—C12	3.6 (4)	C11—C9—C15—O16	−99.6 (3)
C15—C9—C11—C12	−171.5 (3)	O17—C15—O16—C18	−1.1 (5)
C2—C1—C11—C9	−178.2 (3)	C9—C15—O16—C18	179.6 (3)
C2—C1—C11—C12	1.8 (5)	C15—O16—C18—C23	−87.3 (4)
C14—N10—C12—C4	176.3 (3)	C15—O16—C18—C19	97.4 (3)
C25—N10—C12—C4	−5.0 (4)	C23—C18—C19—C20	0.0 (5)
C14—N10—C12—C11	−4.6 (4)	O16—C18—C19—C20	175.2 (3)
C25—N10—C12—C11	174.0 (3)	C18—C19—C20—C21	−0.3 (5)
C3—C4—C12—N10	178.1 (3)	C19—C20—C21—C22	0.1 (5)
C3—C4—C12—C11	−0.9 (5)	C19—C20—C21—C24	−178.9 (3)
C9—C11—C12—N10	0.9 (4)	C20—C21—C22—C23	0.3 (5)
C1—C11—C12—N10	−179.1 (3)	C24—C21—C22—C23	179.3 (3)
C9—C11—C12—C4	180.0 (3)	C19—C18—C23—C22	0.4 (5)
C1—C11—C12—C4	0.0 (4)	O16—C18—C23—C22	−174.8 (3)
C11—C9—C13—C8	176.4 (3)	C21—C22—C23—C18	−0.5 (5)
C15—C9—C13—C8	−8.6 (4)	O27—S26—C30—F32	58.5 (3)
C11—C9—C13—C14	−4.4 (4)	O28—S26—C30—F32	178.4 (3)
C15—C9—C13—C14	170.6 (3)	O29—S26—C30—F32	−62.3 (3)
C7—C8—C13—C9	−179.4 (3)	O27—S26—C30—F31	−61.3 (3)
C7—C8—C13—C14	1.3 (5)	O28—S26—C30—F31	58.6 (3)
C12—N10—C14—C5	−175.6 (3)	O29—S26—C30—F31	177.9 (3)
C25—N10—C14—C5	5.7 (4)	O27—S26—C30—F33	178.2 (3)
C12—N10—C14—C13	3.8 (4)	O28—S26—C30—F33	−61.8 (3)
C25—N10—C14—C13	−174.8 (3)	O29—S26—C30—F33	57.5 (3)
C6—C5—C14—N10	178.8 (3)		

### Hydrogen-bond geometry (Å, °)

**Table:**

*Cg*4 is the centroid of the C18–C23 ring.

**Table:**

*D*—H···*A*	*D*—H	H···*A*	*D*···*A*	*D*—H···*A*
C3—H3···O27^i^	0.93	2.57	3.314 (5)	137
C4—H4···O29^i^	0.93	2.44	3.319 (4)	159
C5—H5···O28	0.93	2.44	3.364 (5)	171
C6—H6···O28^ii^	0.93	2.56	3.342 (5)	142
C23—H23···O27^iii^	0.93	2.53	3.448 (4)	169
C25—H25*A*···O29	0.96	2.56	3.415 (5)	149
C25—H25*B*···*Cg*4^iv^	0.96	2.62	3.487 (4)	151

Symmetry codes:  (i) −*x*+3/2, *y*+1/2, −*z*+1/2; (ii) −*x*+2, −*y*+1, −*z*+1; (iii) *x*−1, *y*, *z*; (iv) −*x*+1, −*y*+1, −*z*+1.

### Table 2 C–F···π interactions (Å,°).

**Table:**

*X*	*I*	*J*	*I*···*J*	*X*···*J*	*X*–*I*···*J*
C30	F31	*Cg*2^i^	3.420 (3)	4.044 (4)	108.9 (2)
C30	F32	*Cg*1^i^	3.441 (3)	4.032 (4)	107.1 (2)
C30	F32	*Cg*2^i^	3.788 (4)	4.044 (4)	91.5 (2)
C30	F33	*Cg*1^i^	3.669 (3)	4.032 (4)	96.2 (2)

Symmetry code: (i) –*x* + 3/2, *y* + 1/2, –*z* + 1/2. Notes: *Cg*1 and *Cg*2 are the centroids of the C9/N10/C11–C14 and C1–C4/C11/C12 rings, respectively.

### Table 3 π–π interactions (Å,°).

**Table:**

*I*	*J*	*CgI*···*CgJ*	Dihedral angle	*CgI*_Perp	Cg*I*_Offset
3	4^v^	3.913 (2)	4.80 (17)	3.472 (2)	1.805 (2)
4	3^v^	3.913 (2)	4.80 (17)	3.565 (2)	1.613 (2)

Symmetry code: (v) –*x* + 1, –*y*, –*z* + 1.Notes: *Cg*3 and *Cg*4 are the centroids of the C5–C8/C13/C14 and C18–C23 rings, respectively. *CgI*···*CgJ* is the distance between ring centroids. The dihedral angle is that between the planes of the rings *I* and *J*. *CgI*_Perp is the perpendicular distance of *CgI* from ring *J*. Cg*I*_Offset is the distance between *CgI* and perpendicular projection of *CgJ* on ring *I*.
